# Production of Induced Secondary Metabolites by a Co-Culture of Sponge-Associated Actinomycetes, *Actinokineospora* sp. EG49 and *Nocardiopsis* sp. RV163

**DOI:** 10.3390/md12053046

**Published:** 2014-05-22

**Authors:** Yousef Dashti, Tanja Grkovic, Usama Ramadan Abdelmohsen, Ute Hentschel, Ronald J. Quinn

**Affiliations:** 1 Eskitis Institute for Drug Discovery, Griffith University, Brisbane, QLD 4111, Australia; E-Mails: yousef.dashti@griffithuni.edu.au (Y.D.); t.grkovic@griffith.edu.au (T.G.); 2 Department of Botany II, Julius-von-Sachs Institute for Biological Sciences, University of Würzburg, Julius-von-Sachs-Platz 3, D-97082 Würzburg, Germany; E-Mails: usama.ramadan@uni-wuerzburg.de (U.R.A.); ute.hentschel@uni-wuerzburg.de (U.H.)

**Keywords:** co-cultivation, induced metabolites, sponge-associated actinomycetes, NMR fingerprint, bioactivity

## Abstract

Two sponge-derived actinomycetes, *Actinokineospora* sp. EG49 and *Nocardiopsis* sp. RV163, were grown in co-culture and the presence of induced metabolites monitored by ^1^H NMR. Ten known compounds, including angucycline, diketopiperazine and β-carboline derivatives **1**–**10**, were isolated from the EtOAc extracts of *Actinokineospora* sp. EG49 and *Nocardiopsis* sp. RV163. Co-cultivation of *Actinokineospora* sp. EG49 and *Nocardiopsis* sp. RV163 induced the biosynthesis of three natural products that were not detected in the single culture of either microorganism, namely *N*-(2-hydroxyphenyl)-acetamide (**11**), 1,6-dihydroxyphenazine (**12**) and 5a,6,11a,12-tetrahydro-5a,11a-dimethyl[1,4]benzoxazino[3,2-*b*][1,4]benzoxazine (**13a**). When tested for biological activity against a range of bacteria and parasites, only the phenazine **12** was active against *Bacillus* sp. P25, *Trypanosoma brucei* and interestingly, against *Actinokineospora* sp. EG49. These findings highlight the co-cultivation approach as an effective strategy to access the bioactive secondary metabolites hidden in the genomes of marine actinomycetes.

## 1. Introduction

The search for novel biologically active natural products sourced from marine microbes continues to be an important endeavour fuelled by the emergence of new infections diseases and chemotherapy resistance. Marine-derived actinomycete collections have recently yielded new compounds with not only potent biological activity, but also novel molecular scaffolds, for example salinosporamide A [1] and marinopyrroles A and B [2]. Salinosporamide A was shown to be an irreversible inhibitor of the 20S proteasome and entered clinical trials against multiple myeloma, only three years after its discovery [3]. However, finding new microbial secondary metabolites is becoming difficult, as the rate of rediscovery of known compounds is increasing [4,5]. On the other hand, genomic sequencing has revealed the presence of a large number of putative biosynthetic gene clusters in the genomes of some microorganisms that encode for secondary metabolites that are not seen under classical cultivation conditions [6,7,8]. Different strategies have been proposed to activate these cryptic biosynthetic pathways. Co-fermentation of microorganisms in a single environment is one of the proposed methods to de-silence biosynthetic pathways for the production of new secondary metabolites [7,9,10,11]. Mixed fermentation of two or more microbes can make a competitive environment, which may induce unexpressed pathways and result in the synthesis of bioactive secondary metabolites due to interspecies crosstalk or chemical defence mechanisms [11,12,13].

Examples of the production of induced new natural products by mixed fermentation of marine-sourced microorganisms include a chlorinated benzophenone pestalone [14] sourced from *Pestalotia* sp. strain CNL-365 and marine α-proteobacterium strain CNJ-328, the diterpenoids libertellenones A–D isolated from a co-culture of the same bacterial strain CNJ-328 with the fungus, *Libertella* sp. CNL-52 [15], and cyclic depsipeptides emericellamides A and B isolated from a co-culture of marine-derived fungus *Emericella* sp. (CNL-878) and marine bacterium *Salinispora arenicola* [16]. In this work, we focus on the induced metabolites from the co-cultivation of two sponge-sourced actinomycetes. Several in-house strains were co-cultured and the presence of differential secondary metabolite production monitored by UV-Vis, MS and NMR techniques. Two strains, namely *Actinokineospora* sp. and *Nocardiopsis* sp., when grown in co-culture showed different chemical profiles to that of the mono-cultures and were prioritised for large-scale natural product isolation work.

Members of the genus *Actinokineospora* were isolated from soil, plants [17,18] and marine sponges [19]. Although this genus is not well known for secondary metabolite production, we recently reported two new angucycline-like compounds named actinosporins A (**1**) and B (**2**) from *Actinokineospora* sp. EG49, where actinosporin A displayed anti-parasitic activity against *Trypanosoma brucei brucei* [20]. On the other hand, the genus, *Nocardiopsis*, is frequently isolated from terrestrial, as well as marine environments, including marine sponges [21,22,23]. Members of this genus are prolific producers of a multitude of secondary metabolites with diverse activities [24,25,26,27]. In this study, two sponge-derived actinomycetes were co-cultured in liquid media; these being *Nocardiopsis* sp. RV163 from the Mediterranean sponge *Dysidea avara*, and *Actinokineospora* sp. EG49 from the Red Sea sponge, *Spheciospongia vagabunda*. To the best of our knowledge, this is the first report of induced metabolites from the mixed fermentation of two sponge-associated actinomycetes.

Traditionally, the detection of induced metabolite biosynthesis has relied either on LC-PDA [5,28,29] or LC-PDA-MS [12,15,16,30], methods to monitor the production of the secondary metabolite by comparison of the small molecule profiles of the mono- and co-cultures of microorganisms. However, these analytical techniques are dependent either on the existence of a chromophore (PDA detection) or the ability of a compound to be ionised (MS detection) and might not detect all of the changes of the secondary metabolome between the mono- and co-cultures. In order to further interrogate the existence of the induced change in the secondary metabolome profiles, we used LC-PDA, as well as ^1^H-NMR fingerprinting techniques. Following the detection of the production of the induced metabolites, an isolation process was performed, which led to the identification of *N*-(2-hydroxyphenyl)-acetamide (**11**), 1,6-dihydroxyphenazine (**12**) and 5a,6,11a,12-tetrahydro-5a,11a-dimethyl[1,4]benzoxazino[3,2-*b*][1,4]benzoxazine (**13a**).

## 2. Results and Discussion

**Figure 1:**
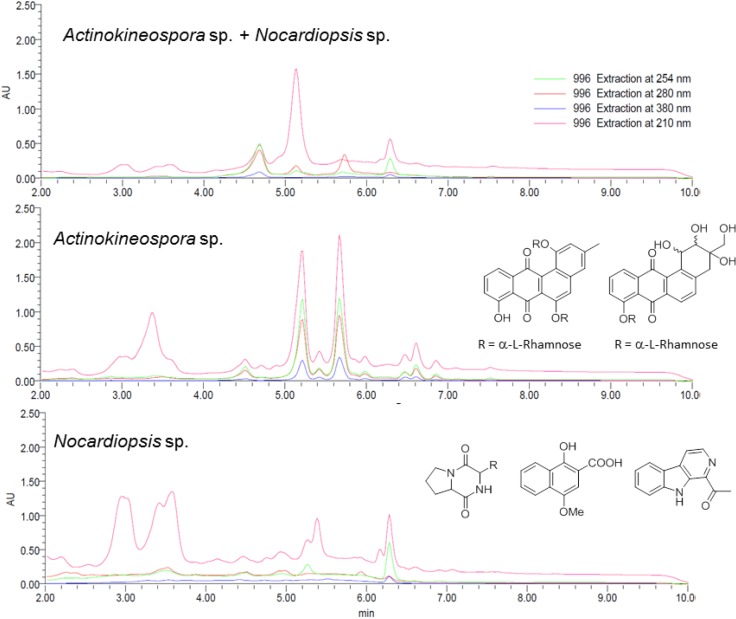
HPLC chromatograms of the EtOAc extracts of *Actinokineospora* sp. EG49 and *Nocardiopsis* sp. RV163 co-culture (**top**), *Actinokineospora* sp. EG49 monoculture (**middle**) and *Nocardiopsis* sp. RV163 monoculture (**bottom**). The depicted chromatograms were extracted at 210, 254, 280 and 380 nm, and the bottom two spectra show representative examples of the natural products isolated from the two strains.

The mono- and co-culture secondary metabolite profiles were monitored with a combination of UV-PDA and NMR-based spectroscopic techniques. Figure 1 depicts the LC-PDA metabolic profile of the three actinomycetes cultures and shows that the co-culture extract displayed a very different chemotype compared to that of the two single cultures. In order to further investigate the differences of the secondary metabolite profiles by ^1^H-NMR and to have a sufficient quantity to identify the metabolites, a large-scale study was undertaken on 50 mg of the EtOAc extract. The methodology utilised identical reversed-phase C_18_ stationary support as for the analytical HPLC run, but allowed for a longer elution gradient, which gave better sensitivity and resolution of the secondary metabolites present. ^1^H-NMR spectra were then used to compare the differences between each chromatography fraction sourced from the mono- and co-cultures.

### 2.1. Monoculture Chemical Profiles

**Figure 2:**
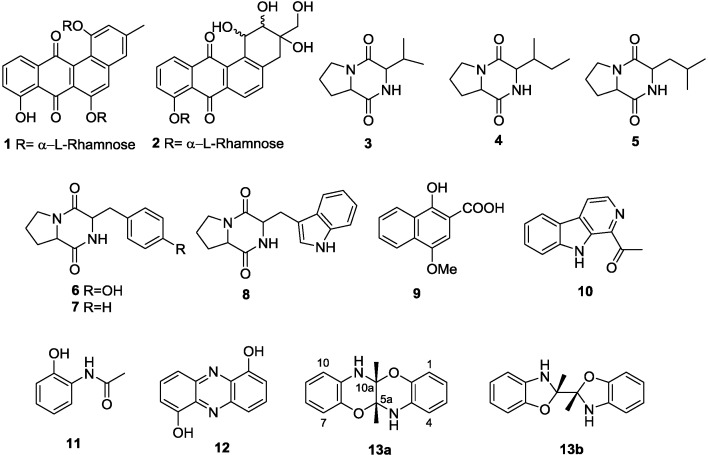
The structure of the major compounds identified from the EtOAc extracts of *Actinokineospora* sp. EG49 actinosporins A (**1**) and B (**2**); *Nocardiopsis* sp. RV163 cyclo-(prolyl-valyl) (**3**), cyclo-(isoleucyl-prolyl) (**4**), cyclo-(leucyl-prolyl) (**5**), cyclo-(prolyl-tyrosyl) (**6**), cyclo-(phenylalanyl-prolyl) (**7**), cyclo-(prolyl-tryptophyl) (**8**), 1-hydroxy-4-methoxy-2-naphthoic acid (**9**) and 1-acetyl-β-carboline (**10**);and the co-culture, *N*-(2-hydroxyphenyl)-acetamide (**11**), 1,6-dihydroxyphenazine (**12**), 5a,6,11a,12-tetrahydro-5a,11a-dimethyl-1,4-benzoxazino[3,2-*b*][1,4]benzoxazine (**13a**) and 2,2′,3,3′-tetrahydro-2,2′-dimethyl-2,2′-bibenzoxazole (**13b**).

Previously, we reported on the structures of two new angucycline-type metabolites, actinosporins A (**1**) and B (**2**) (Figure 2), isolated from *Actinokineospora* sp. EG49 [20]. In this work, compounds **1** and **2** were confirmed to be the major metabolites present in this extract, and further attempts at the structure elucidation of minor metabolites were not made. The majority of the natural products present in the EtOAc extract of *Nocardiopsis* sp. RV163 belonged to the diketopiperazine class of compounds. They were identified as 2,5-diketopiperazines cyclo-(prolyl-valyl) (**3**) [31], cyclo-(isoleucyl-prolyl) (**4**) [32], cyclo-(leucyl-prolyl) (**5**) [31], cyclo-(prolyl-tyrosyl) (**6**) [33], cyclo-(phenylalanyl-prolyl) (**7**) [32] and cyclo-(prolyl-tryptophyl) (**8**) [34,35]. The purity of the compounds (at <90%) prevented us from confirming the absolute configuration of the diketopiperazines, **3**–**8**. Two other secondary metabolites in *Nocardiopsis* sp. RV163 EtOAc extract were identified as known natural products 1-hydroxy-4-methoxy-2-naphthoic acid (**9**) [36] and 1-acetyl-β-carboline (**10**) [37].

### 2.2. Co-Culture Chemical Profile

**Figure 3:**
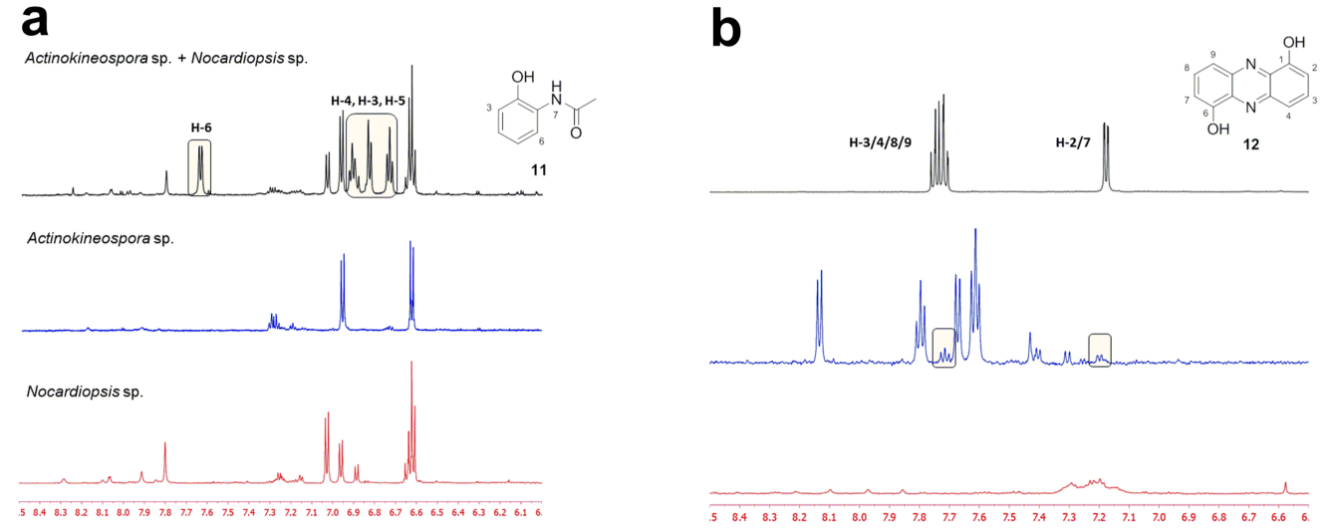
^1^H-NMR fingerprints of HPLC fractions sourced from the EtOAc extracts of *Actinokineospora* sp. EG49 and *Nocardiopsis* sp. RV163 co-culture (**top** in black), *Actinokineospora* sp. EG49 monoculture (**middle** in blue) and *Nocardiopsis* sp. RV163 monoculture (**bottom** in red). (**a**) ^1^H-NMR spectra of fraction 5; chemical shifts for the induced metabolite *N*-(2-hydroxyphenyl)-acetamide (**11**) are highlighted;(**b**) ^1^H-NMR fingerprints of fraction 24; the possible presence of compound **12** in the *Actinokineospora* sp. EG49 monoculture is highlighted.

Having established the UV-PDA, MS and the ^1^H-NMR profile of the two monocultures, the co-culture extract was investigated. The ^1^H-NMR spectra of the same chromatography fractions of mono- and co-culture extracts were compared, and since the retention times of compounds can vary, the neighbouring fractions were also considered. The presence of the first induced metabolite was apparent in the ^1^H-NMR spectra of fraction 5 of co-culture compared to that of the mono-cultures. Aromatic signals in the region 6.70 to 7.70 ppm were observed in co-culture that were not present in the spectra of the *Actinokineospora* sp. EG49- and *Nocardiopsis* sp. RV163-sourced fractions (Figure 3a). The absence of these NMR signals in both monocultures suggested that this was an induced metabolite produced through mixed fermentation of the two actinomycetes. A literature search based on the molecular ion and structural information generated from the ^1^H-NMR spectrum identified this compound to be the known natural product, *N*-(2-hydroxyphenyl)-acetamide (**11**) [38].

In addition to fraction 5, two other chromatography fractions showed the presence of induced metabolites. In regions concentrated around fraction 24, aromatic signals between 7.15 and 7.80 ppm and an exchangeable one at 10.45 ppm were detected in co-culture, and while initially this compound was not apparent in either of the two monocultures, the ^1^H-NMR fingerprint suggested that this metabolite may be present in very small amounts in the *Actinokineospora* sp. EG 49 extract (Figure 3b). In order to further interrogate whether compound **12** was present in the *Actinokineospora* sp. EG49 monoculture extract, we performed an NMR titration experiment (see Supplementary Information). NMR-detected titration of pure **12** into fraction 24 sourced from the *Actinokineospora* sp. EG49 extract demonstrated that compound **12** was not present in the mono-culture sample and confirmed that this small molecule is another induced metabolite from the mixed fermentation experiment. Based on the comparison of the mass and the NMR information with the literature, the induced metabolite was identified to be 1,6-dihydroxyphenazine (**12**) [39]. Compound **12** was produced in a very high yield (12% crude weight), and being in such a high abundance we found that this major component was present in the NMR fingerprints of many of the consequent fractions of the co-culture extract. Fractions 26 and 27, in addition to having ^1^H-NMR signals of 1,6-dihydroxyphenazine, showed additional proton signals in the aromatic region, which were not present in either of the monoculture extracts (for the NMR fingerprint comparison, see Supplementary Information). The spectroscopic and spectrometric data of the third induced metabolite were consistent with the structures of 5a,6,11a,12-tetrahydro[1,4]benzoxazino[3,2-*b*][1,4]benzoxazine (**13a**) and 2,2′,3,3′-tetrahydro-2,2′-dimethyl-2,2′-bibenzoxazole (**13b**) (Figure 2). Both compounds have previously been reported as the condensation product of 2-propyn-1-ol and dibenzo-2-amino alcohol, with the *cis*-fused benzoxazino-benzoxazine structure, **13a**, confirmed as the correct product via single crystal X-ray analysis [40]. In this work, an insufficient amount of the compound has been isolated to provide a good quality crystal for an X-ray, and the assignment was based on NMR data. However, in contrast to [2,2′]bifuranyl-pyranopyran models [41], the ^13^C chemical shifts of the ring fusion atoms in **13a** and **13b** do not provide the distinction of the ring size [42]. Based on the literature evidence of compound **13a** being the major isomer in solution [43], we propose the structure of the induced natural product to be 5a,6,11a,12-tetrahydro-5a,11a-dimethyl[1,4]benzoxazino[3,2-*b*][1,4]benzoxazine (**13a**). Since this is the first report of compound **13a** as a natural product, a complete set of NMR data is given in pyridine-*d*_5_.

In addition to tracking the production of the induced metabolites in the co-culture extract, the NMR experiment was also useful in showing the suppression of the production of some natural products. For example, while in Figure 3b, the main compound in fraction 24 sourced from the *Actinokineospora* sp. EG49 crude extract is actinosporin B (**2**) (blue, middle spectrum), in the co-culture NMR spectrum (black, top spectrum), all resonances for compound **2** are missing, indicating that the mixed fermentation suppressed the production of actinosporin B. In support of the NMR observations, the mixed culture of the two microbes was dominated by the *Nocardiopsis* sp. RV163, which is characterized by large brown colonies and the production of spores, while very little growth was observed by the yellow cells of the *Actinokineospora* sp. EG49.

### 2.3. Anti-Infective Activity of Induced Metabolites

The three induced compounds, *N*-(2-hydroxyphenyl)-acetamide (**11**), 1,6-dihydroxyphenazine (**12**) and 5a,6,11a,12-tetrahydro-5a,11a-dimethyl[1,4]benzoxazino[3,2-*b*][1,4]benzoxazine (**13a**), were tested for their activities against *Bacillus* sp. P25, *Escherichia coli* and *Fusarium* sp. P21, human parasites *Leishmania major* and *Trypanosoma brucei*, as well as *Nocardiopsis* sp. RV163 and *Actinokineospora* sp. EG49 cultures. Biological activity was documented for compound **12** against *Bacillus* sp. (11 mm inhibition zone diameter), *Trypanosoma brucei* (IC_50_ value of 19 μM) and, interestingly, against *Actinokineospora* sp. EG49 (15 mm inhibition zone diameter) (see Supplementary Information).

## 3. Experimental Section

### 3.1. General Experimental Procedures

Optical rotations were measured on a JASCO P-1020 polarimeter with a 10-cm cell. UV spectra were acquired on a Jasco V650 UV/vis spectrophotometer. A Jasco J-715 spectropolarimeter was used to record circular dichroism spectra. NMR spectra were recorded at 30 °C on a Varian Inova 600 MHz spectrometer equipped with a triple resonance 5-mm cold probe. For NMR fingerprint experiments, the samples were dissolved in 230 μL of DMSO-*d*_6_ and run in a 3-mm NMR tube. The standard VnmrJ 3.2 Proton pulse sequence was run with the following parameters: pw = 45°, p1 = 0 μs, d2 = 0 s, d1 = 1 s, at = 1.7 s, sw = 9615 Hz, nt = 8 scans. LC-MS spectra were obtained using a Waters ZQ electrospray mass spectrometer with a Phenomenex Luna C_18_ column (4.6 mm × 50 mm, 3 μm) (Phenomenex, Torrance, CA, USA). Analytical HPLC was done with a Phenomenex Onyx Monolithic C_18_ column (4.6 × 100 mm) (Phenomenex, Torrance, CA, USA). A Phenomenex Onyx Monolithic C_18_ column (10 mm × 100 mm) (Phenomenex, Torrance, CA, USA) was used for semi-preparative HPLC separation. All HPLC and LC-MS experiments were performed with a MeOH-H_2_O gradient solvent system. Millipore Milli-Q PF filtered H_2_O and HPLC grade solvents were used for chromatography.

### 3.2. Microbial Fermentation and Extracts Preparation

*Nocardiopsis* sp. RV163 was isolated from the Mediterranean sponge, *Dysidea avara*, while *Actinokineospora* sp. EG49 was cultivated from the Red Sea sponge, *Spheciospongia vagabunda* [19]. Each strain was fermented in 8 Erlenmeyer flasks (2 L), each containing 1 L of ISP 2 (International Streptomyces Project) medium in artificial sea water and incubated at 30 °C for 7 days with shaking at 150 rpm. For co-cultivation experiment, 10 mL of 5-day-old culture of *Nocardiopsis* sp. RV163 was inoculated into 8 Erlenmeyer flasks (2 L), each containing 1 L of ISP 2 medium inoculated with 10 mL of 5-day-old culture of *Actinokineospora* sp. EG49. After fermentation of single cultures and co-culture, filtration was done, and the supernatant was extracted with ethyl acetate (2 × 500 mL) to give the ethyl acetate extract. XAD16 resin was then added to the mother liquor, shaken, filtered and finally extracted with acetone (acetone extract). The cells and mycelia were macerated in a double volume of methanol with shaking for 3 h, then filtered (methanolic extract).

### 3.3. Extraction and Isolation

#### 3.3.1. General Chromatographic Procedures for Large-Scale Isolation and Fingerprinting Work

Crude extract (50 mg) was chromatographed using HPLC on a semi-preparative Phenomenex Onyx Monolithic reversed-phase C_18_ column (10 mm × 100 mm). Initially, isocratic conditions of 10% MeOH were used for 10 min, then a linear gradient from 10% to 100% MeOH was performed over 40 min and continued isocratically for 10 min at a flow rate of 9 mL/min. Sixty fractions collected in one minute increments over 60 min were dried for NMR and mass studies.

#### 3.3.2. *Actinokineospora* sp. EG49

EtOAc extract (50 mg) was pre-adsorbed to C_18_-bonded silica and then packed into a stainless steel HPLC guard cartridge (10 × 30 mm) that was subsequently attached to a C_18_ HPLC column. Standard gradient conditions described above were employed to give actinosporin B (**10**) in fraction 24 and actinosporin A (**1**) in fraction 31.

#### 3.3.3. *Nocardiopsis* sp. RV163

EtOAc extract (50 mg) was pre-adsorbed to C_18_-bonded silica and then packed into a stainless steel HPLC guard cartridge (10 × 30 mm) that was subsequently attached to a C_18_ HPLC column. Standard gradient conditions described above were employed. Fraction 9 was a mixture of cyclo-(prolyl-valyl) (**3**), cyclo-(isoleucyl-prolyl) (**4**) and cyclo-(leucyl-prolyl) (**5**). Semi-pure cyclo-(prolyltyrosyl) (**6**) (1.6 mg) and cyclo-(phenylalanyl-prolyl) (**7**) (1.0 mg) were identified from fractions 15 and 16, respectively, cyclo (prolyl-tryptophyl) (**8**) was identified from fraction 17. 1-hydroxy-4-methoxy-2-naphthoic acid (**9**) (0.7 mg) and 1-acetyl-β-carboline (**10**) (0.2 mg) were identified in fractions 26, 27 and 30, respectively.

#### 3.3.4. *Actinokineospora* sp. EG49 and *Nocardiopsis* sp. RV163 Co-Culture

EtOAc extract (50 mg) was pre-adsorbed to C_18_-bonded silica and then packed into a stainless steel HPLC guard cartridge (10 × 30 mm) that was subsequently attached to a C_18_ HPLC column. Standard gradient conditions described above were employed to give *N*-(2-hydroxyphenyl)-acetamide (**11**) (0.3 mg) purified from fraction 5; diketopiperazines (**3**–**8**) were eluted in the same fractions as the mono-culture extract of *Nocardiopsis* sp.; the major induced compound, 1,6-dihydroxyphenazine (**12**) (5.9 mg), started to elute in fraction 24 and continued to elute off the column until fraction 42. Fractions 25 to 28 were combined and re-run on a reverse-phase HPLC using Phenomenex Onyx Monolithic C_18_ column (10 mm × 100 mm) eluting with a gradient from H_2_O/MeOH (80:20 to 40:60 over 60 min) to purify compound **13a** (0.6 mg).

### 3.4. Bioactivity Testing

#### 3.4.1. Antibacterial Activity

The induced compounds **11**, **12** and **13a** were tested for their antimicrobial activity using the standard disk diffusion assay against *Bacillus* sp. P25, *Escherichia coli* and *Fusarium* sp. P21, as well as the actinomycetes from where the compounds were derived, *Nocardiopsis* sp. RV163 and *Actinokineospora* sp. EG49. Sterile filter disks (6-mm diameter) loaded with the test compounds (25 μL of 1 mg/mL in methanol) were placed on agar plates that had been inoculated with 100 μL of the test microorganism (cultures with an optical density of OD_600_ = 0.2). After incubation (24 h for *Bacillus*, *Escherichia coli* and *Fusarium* sp. and 72 h for *Nocardiopsis* sp. RV163 and *Actinokineospora* sp. EG49) at 37 °C (*Bacillus*, *Escherichia coli*) and 30 °C (*Fusarium* sp., *Nocardiopsis* sp. RV163 and *Actinokineospora* sp. EG49), the antimicrobial potential was quantitatively assessed as the diameter of the inhibition zone (*n* = 2).

#### 3.4.2. Anti-Trypanosomal Activity

Anti-trypanosomal activity was tested following the protocol of Huber and Koella [44]. In complete Baltz medium, 10^4^ trypanosomes per millilitre of *Trypanosoma brucei* strain TC 221 were cultivated. Trypanosomes were tested in 96-well plate chambers against different concentrations of test substances at 0.25–40 μM in 1% DMSO to a final volume of 200 μL*.* For controls, 1% DMSO, as well as parasites without any test compounds were used simultaneously in each plate to show no effect of 1% DMSO. The plates were then incubated at 37 °C in an atmosphere of 5% CO_2_ for 24 h. After the addition of 20 μL of Alamar Blue, the activity was measured after 48 and 72 h by light absorption using an MR 700 Microplate Reader at a wavelength of 550 nm with a reference wavelength of 650 nm. The IC_50_ value effect of the test compound was quantified by the linear interpolation of three independent measurements.

The following equation was used to calculate IC_50_:

log(IC_50_) = log(X_1_) + {[(Y_1_ − 0.5)/(Y_1_ − Y_2_)] × [log(X_2_) − log(X_1_)]}

Y_1_ mean of the duplicate determination of the first measured cell density that is less than half the average of the growth control divided by the average of control growth.
X_1_ concentration of the substance that belongs to the cell density of Y_1_.
Y_2_ mean of the duplicate determination of the first measured cell density that is greater than half the average of the growth control divided by the average of control growth.
X_2_ concentration of the substance that belongs to the cell density of Y_2_.

#### 3.4.3. Anti-Leishmanial Activity

Anti-leishmanial activity was tested following the method of Ponte-Sucre *et al.* [45]. Briefly, 10^7^ cells/mL *Leishmania major* promastigotes were incubated in complete medium for 24 h at 26 °C, 5% CO_2_ and 95% humidity in the absence or presence of different concentrations of the test compounds (0.25–40 μM in 1% DMSO) to a final volume of 200 μL. Following the addition of Alamar Blue, the plates were incubated again, and the optical densities were determined after 48 h with a Multiskan Ascent enzyme-linked immunosorbent assay (ELISA) reader (Multiskan Ascent, Germany). The effects of cell density, incubation time and the concentration of DMSO were examined in control experiments. The results were expressed in IC_50_ values by linear interpolation of three independent experiments.

### 3.5. Structure Elucidation of Compounds **1**–**13**

The structures of all known compounds were confirmed upon comparison of spectrometric (low-resolution MS) and spectroscopic (^1^H, ^13^C and 2D, where necessary) data with that of the published literature values.

5a,6,11a,12-tetrahydro-5a,11a-dimethyl[1,4]benzoxazino[3,2-*b*][1,4]benzoxazine (**13a**): Yellow oil; 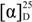 = 0 (*c* 0.012, MeOH); UV (MeOH) λ_max_ (log ε), 295 (3.61), 235 (3.77), 208 (4.36) nm; ^1^H, ^13^C NMR data in DMSO-*d*_6_ in good agreement with published values; ^1^H-NMR (pyridine-*d*_5_, 600 MHz) δ (*J* in Hz) 1.75 (s, 2CH_3_), 6.80 (t, 7.6, H-3/9), 6.90 (t, 7.6, H-2/8), 6.98 (d, 2H, H-4/10), 7.03 (d, 2H, H-1/7), 8.00 (s, 2NH); ^13^C NMR (pyridine-*d*_5_, 125 MHz) 22.2 (2CH_3_), 83.5 (C-5a/11a), 115.5 (C-1/7), 117.6 (C-4/10), 120.2 (C-3/9), 121.9 (C-2/8), 131.8 (C-6a/12a), 143.5 (C-4a/10a), LRESIMS *m/z* 269.3 [M + H]^+^.

## 4. Conclusions

The genomes of microorganisms, particularly of the order, Actinomycetales, consist of a large number of putative biosynthetic gene clusters that encode for secondary metabolites that are not produced using standard fermentation protocols. To reach this cryptic treasure trove of natural products, approaches, such as co-cultivation, are required to induce them. In this study, two sponge-derived actinomycetes, *Nocardiopsis* sp. RV163 and *Actinokineospora* sp. EG49, were co-fermented in liquid media. The presence of induced metabolites was studied by comparison of the ^1^H-NMR fingerprints of the crude extracts of the two monocultures and the co-culture. The NMR fingerprint allowed the confidence of detecting all small molecules containing proton nuclei and was instrumental in demonstrating that the induced metabolites were co-culture specific and not present in either of the single cultures. Co-cultivation of *Nocardiopsis* sp. RV163 and *Actinokineospora* sp. EG49 induced the biosynthesis of three compounds, which were not detected in either microorganism in a single culture, namely *N*-(2-hydroxyphenyl)-acetamide (**11**), 1,6-dihydroxyphenazine (**12**) and 5a,6,11a,12-tetrahydro-5a,11a-dimethyl[1,4]benzoxazino[3,2-*b*][1,4]benzoxazine (**13a**). When tested for biological activity against a range of bacteria and parasites, only the phenazine, **12**, was active against *Bacillus* sp. P25, *Trypanosoma brucei* and interestingly against *Actinokineospora* sp. EG49. Moreover, while not detectable by ^1^H-NMR in the monocultures, compound **12** was produced in a very high yield (12% crude weight) by the co-culture of the two microbes. These findings highlight the co-cultivation approach as an effective strategy to increase the yield of metabolites undetected in the single microbial culture and enhance the chemical diversity of the secondary metabolites hidden in the genomes of marine actinomycetes.

## Supplementary Files

Supplementary File 1 Supplementary Information (PDF, 685 KB)
