# Supplementary material for: Production of Induced Secondary Metabolites by a Co-Culture of Sponge-Associated Actinomycetes, Actinokineospora sp. EG49 and Nocardiopsis sp. RV163
Source: Mar Drugs. 2014 May 22;12(5):3046–59. doi: 10.3390/md12053046 (PMC4052330; doi:10.3390/md12053046)
Supplement: Supplementary File 1 — Supplementary Information (PDF, 685 KB) [file marinedrugs-12-03046-s001.pdf]

Supplementary Information

Figure S1. ^{1}H-NMR spectrum of *N*-(2-hydroxyphenyl)-acetamide (**11**) in DMSO-d_6.

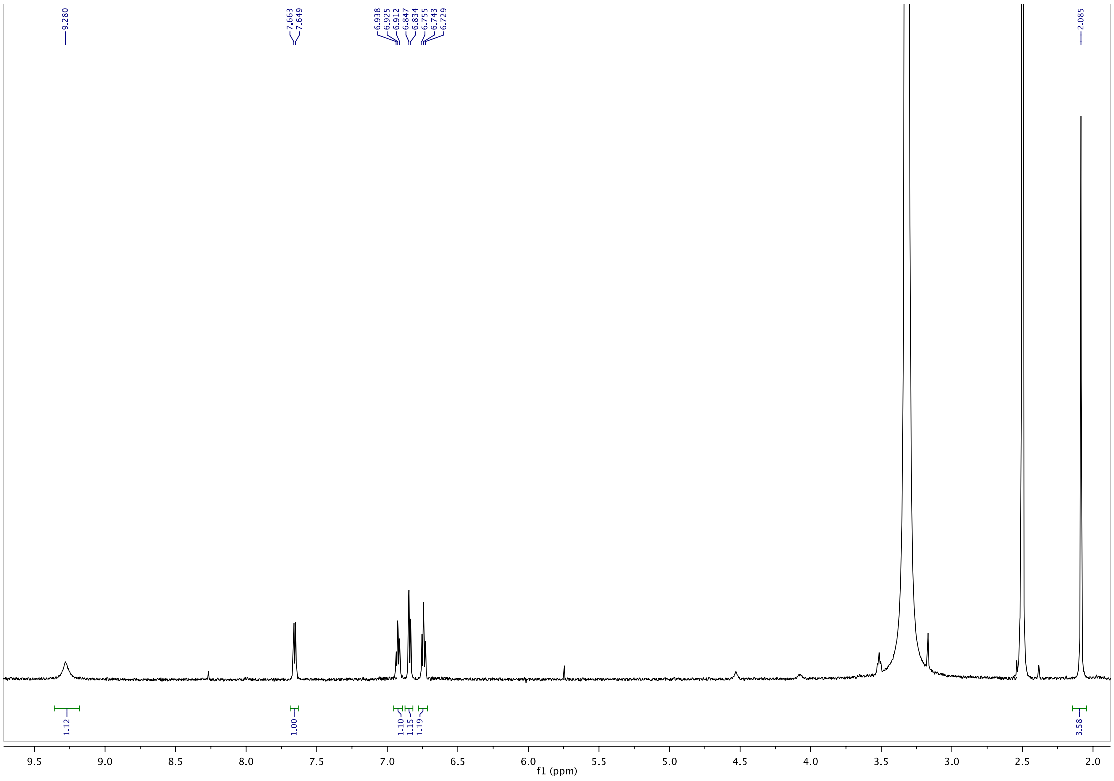

Figure S2. ^{1}H-NMR spectrum of 1,6-dihydroxyphenazine (**12**) in DMSO-d_6.

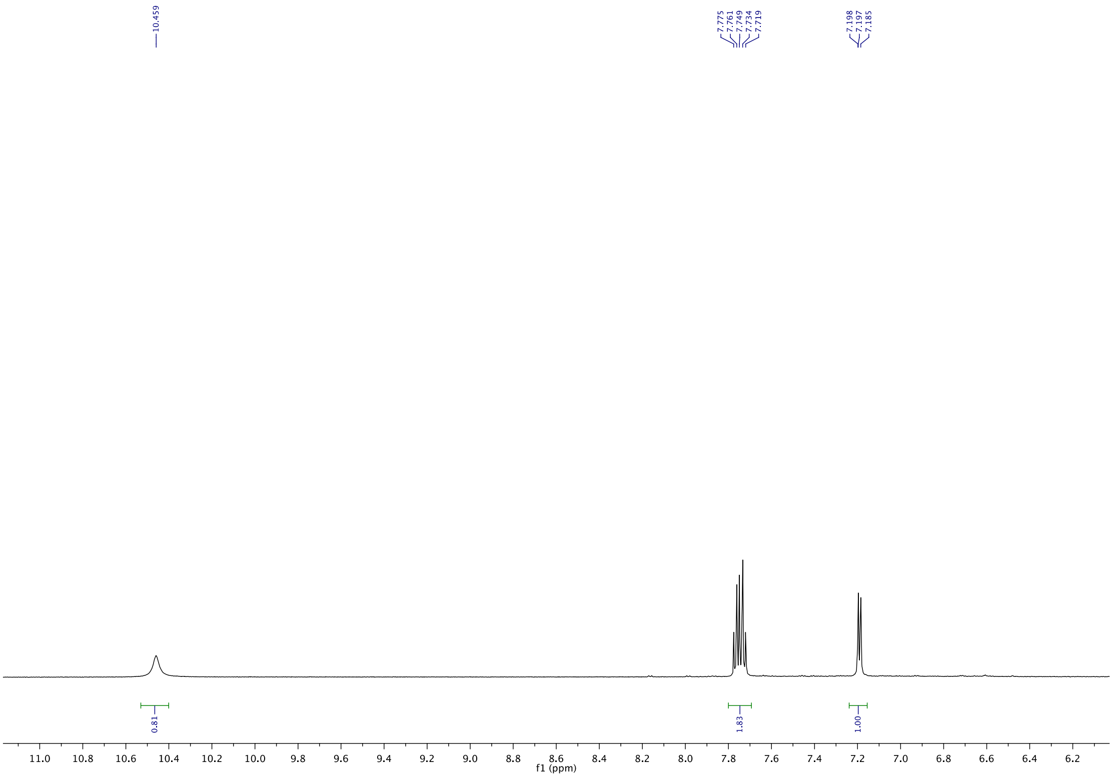

Figure S3. ^{1}H-NMR spectrum of 5a,6,11a,12-tetrahydro-5a,11a-dimethyl[1,4]benzoxazino [3,2-*b*][1,4]benzoxazine (**13a**) in DMSO-d_6.

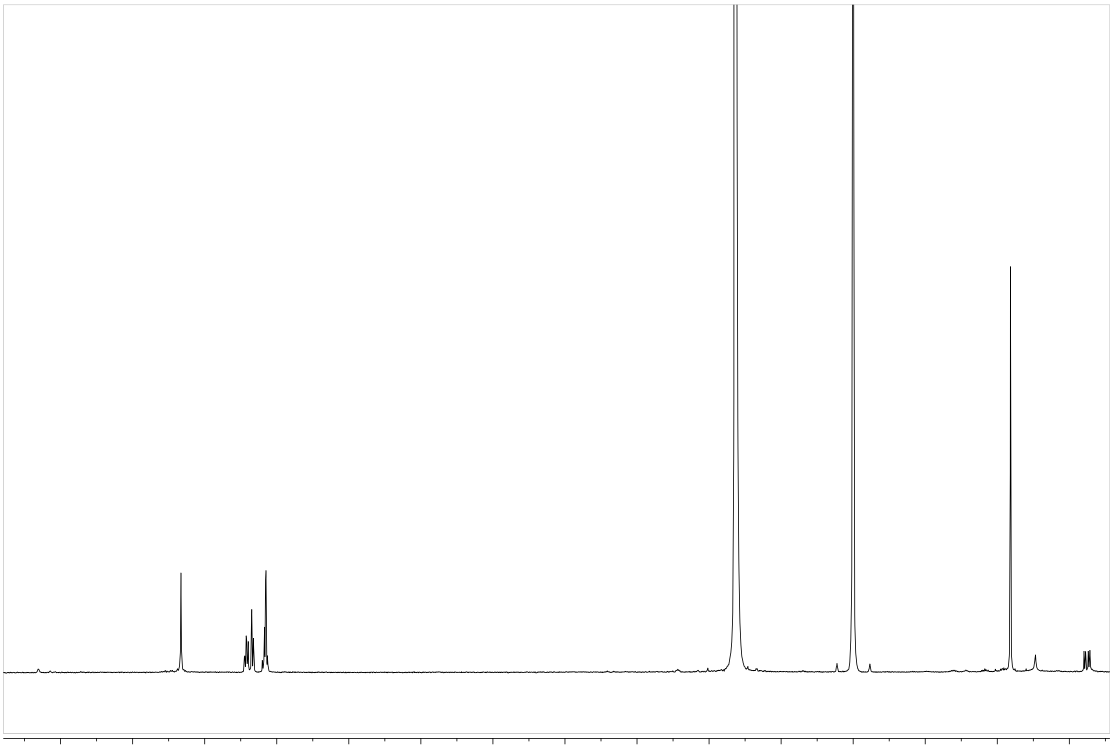

Figure S4. ^{13}C-NMR spectrum 5a,6,11a,12-tetrahydro-5a,11a-dimethyl[1,4]benzoxazino [3,2-*b*][1,4]benzoxazine (**13a**) in DMSO-d_6.

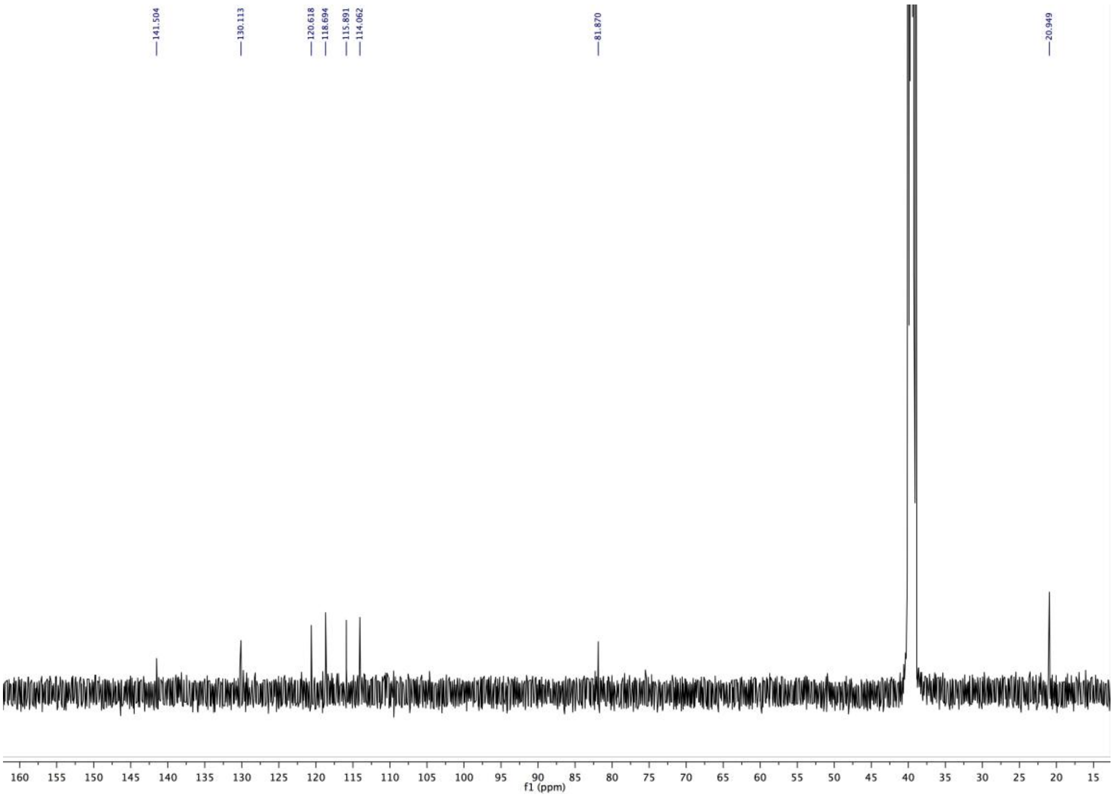

Figure S5. ^{1}H-NMR spectrum of 5a,6,11a,12-tetrahydro-5a,11a-dimethyl[1,4]benzoxazino [3,2-*b*][1,4]benzoxazine (**13a**) in pyridine-d_5.

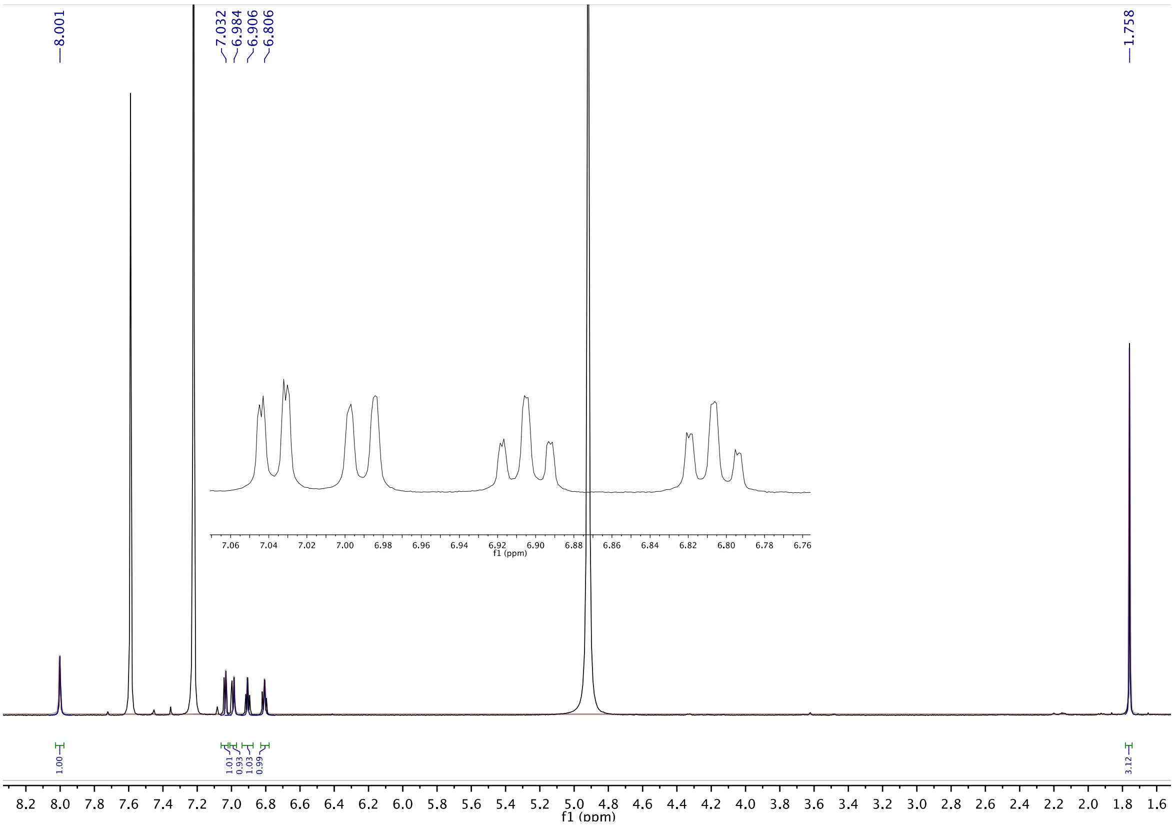

Figure S6. ^{13}C-NMR spectrum of 5a,6,11a,12-tetrahydro-5a,11a-dimethyl[1,4] benzoxazino[3,2-*b*][1,4]benzoxazine (**13a**) in pyridine-d_5.

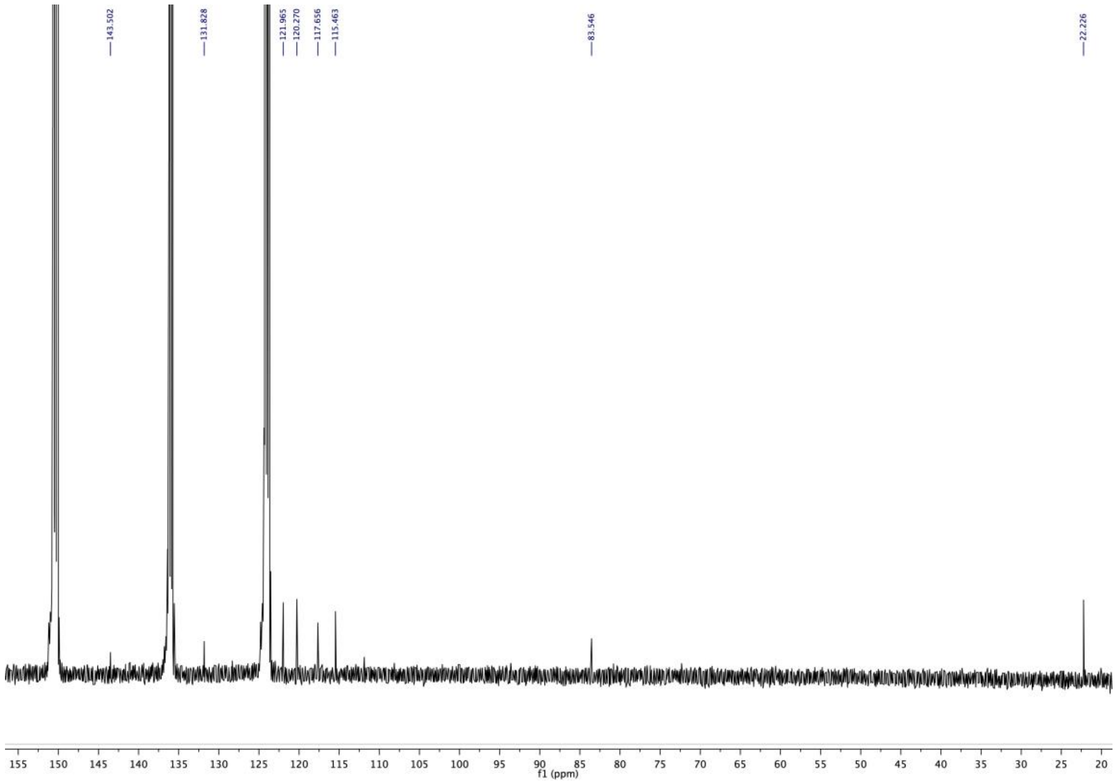

Figure S7. Edited gHSQC spectrum of 5a,6,11a,12-tetrahydro-5a,11a-dimethyl[1,4] benzoxazino[3,2-*b*][1,4]benzoxazine (**13a**) in pyridine-d_5.

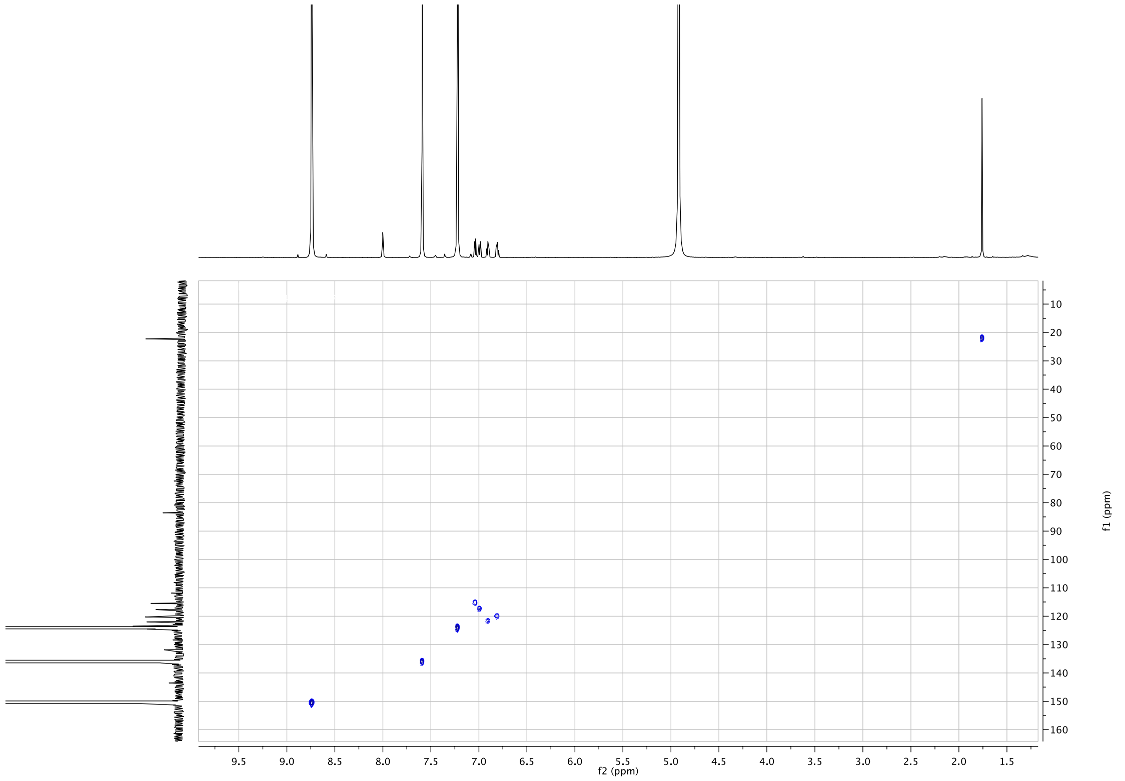

Figure S8. HMBC spectrum of 5a,6,11a,12-tetrahydro-5a,11a-dimethyl[1,4]benzoxazino [3,2-*b*][1,4]benzoxazine (**13a**) in pyridine-d_5.

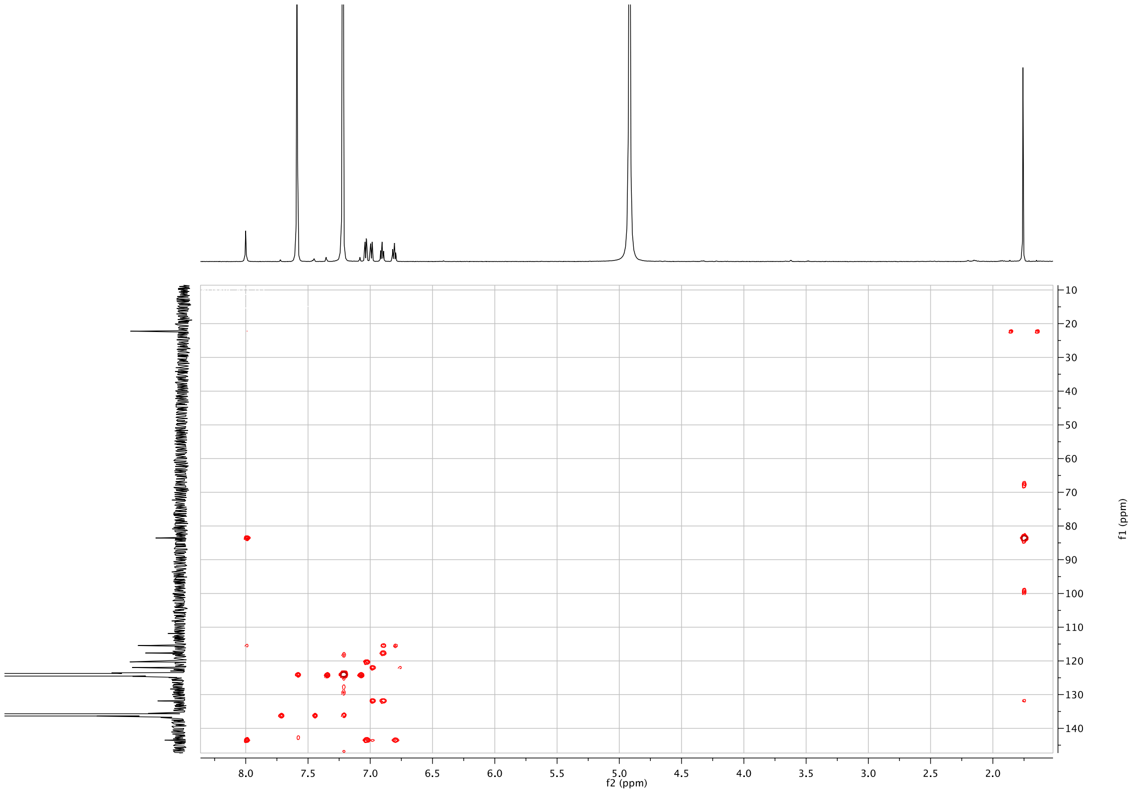

Figure S9. NMR titration of 1,6-dihydroxyphenazine (**12**) in fraction 24 sourced from *Actinokineospora* sp. EG49 in DMSO-d_6.

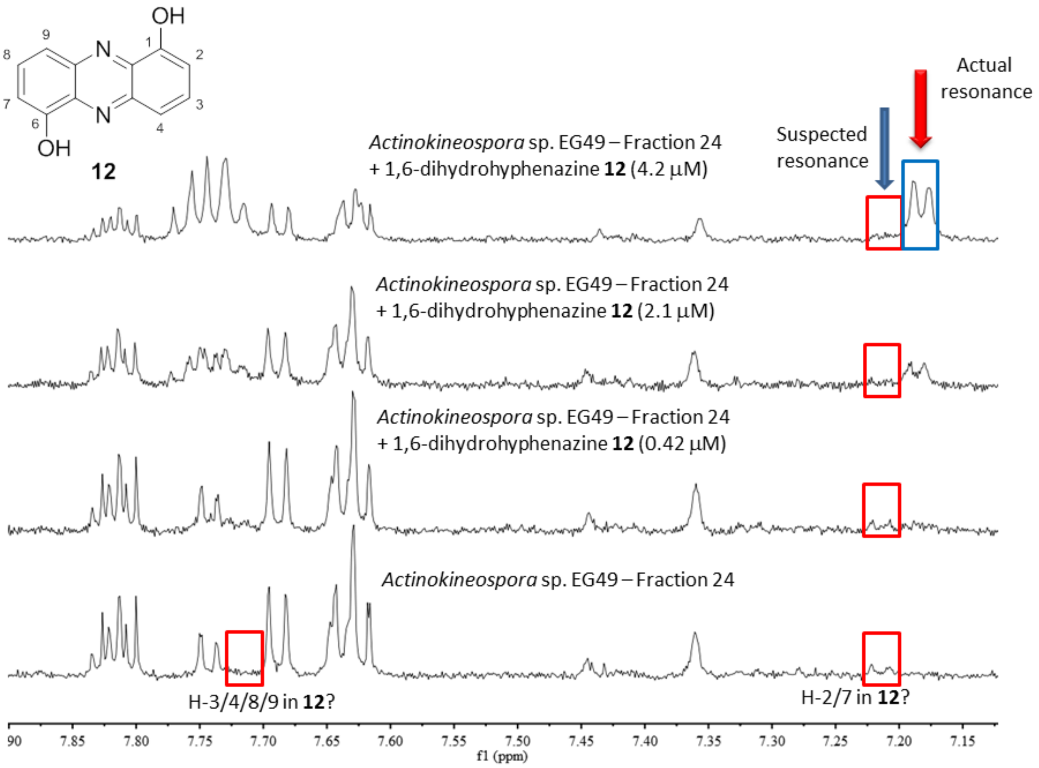

Figure S10. ^{1}H NMR fingerprints of HPLC fractions 26 (**A**) and 27 (**B**) sourced from the EtOAc extracts of the *Nocardiopsis* sp. and *Actinokineospora* sp. co-culture (black) and the *Actinokineospora* sp. monoculture (blue) and *Nocardiopsis* sp. monoculture (red).

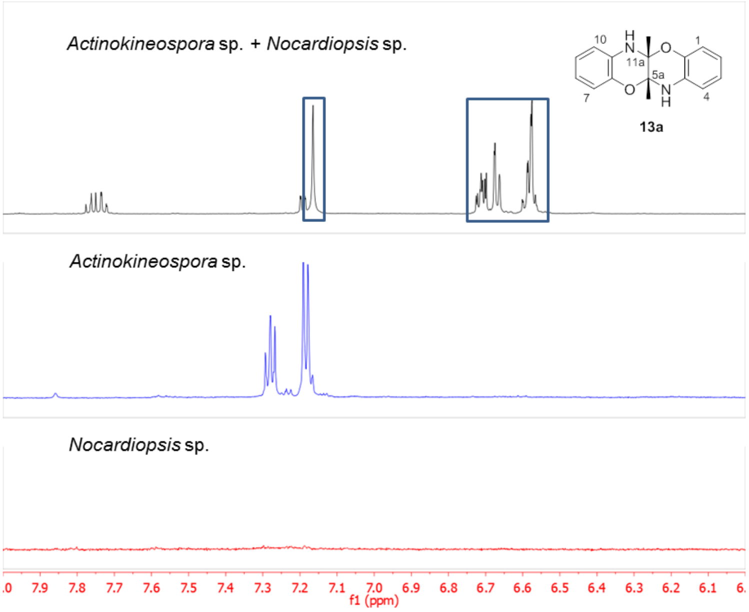

(A)

Figure S10. *Cont.*

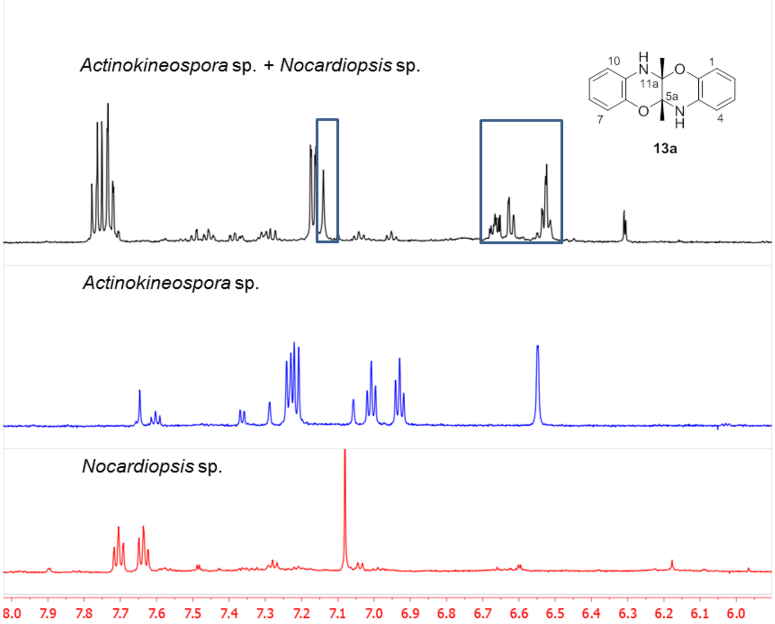

(**B**)

Figure S11. Biological activity of compounds **11**, **12** and **13** against *Bacillus* sp. P21, with the white arrow showing the inhibition zone diameter.

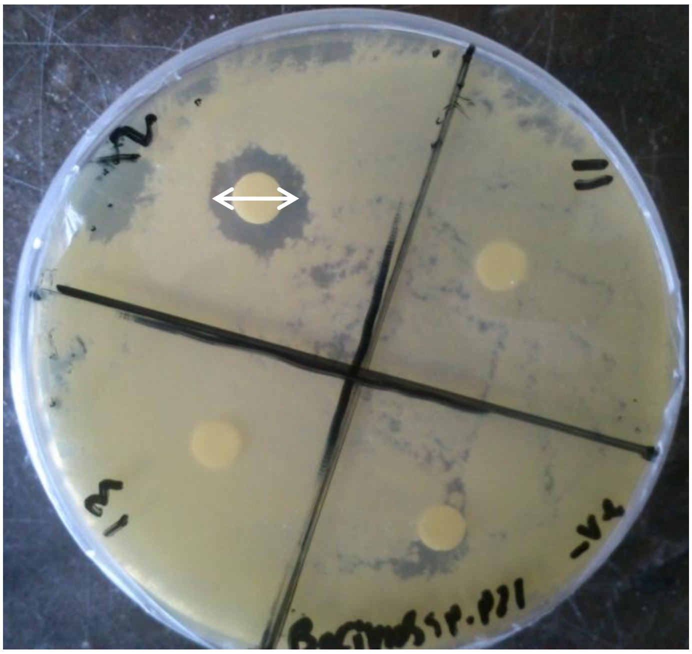

Figure S12. Biological activity of compounds **11**, **12** and **13** against *Actinokineospora* sp. EG49, with the white arrow showing the inhibition zone diameter.

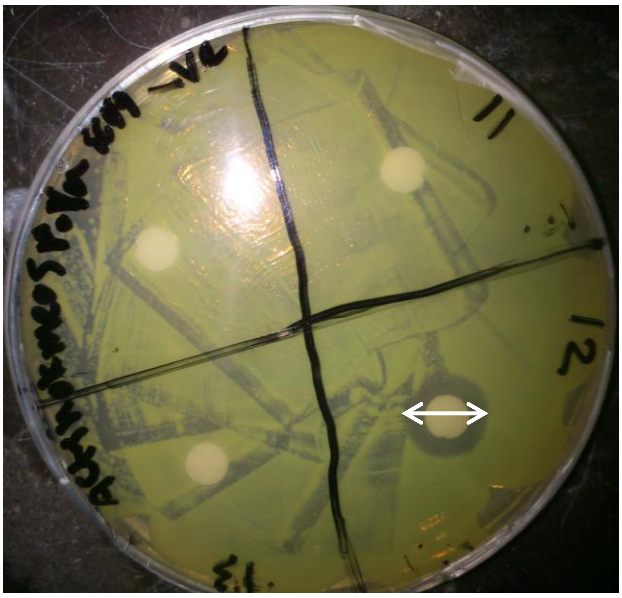

© 2014 by the authors; licensee MDPI, Basel, Switzerland. This article is an open access article distributed under the terms and conditions of the Creative Commons Attribution license (http://creativecommons.org/licenses/by/3.0/).
